# A global cross-cultural analysis of string figures reveals evidence of deep transmission and innovation

**DOI:** 10.1098/rsif.2024.0673

**Published:** 2024-12-04

**Authors:** Roope O. Kaaronen, Matthew J. Walsh, Allison K. Henrich, Isobel Wisher, Elena Miu, Mikael A. Manninen, Jussi T. Eronen, Felix Riede

**Affiliations:** ^1^ PAES Research Unit, Faculty of Biological and Environmental Sciences, University of Helsinki, Helsinki, Finland; ^2^ Modern History and World Cultures Section, The National Museum of Denmark, Copenhagen, Denmark; ^3^ College of Science and Engineering, Seattle University, Seattle, WA, USA; ^4^ Department of Archaeology and Heritage Studies, Aarhus University, Aarhus, Denmark; ^5^ BIOS Research Unit, Helsinki, Finland

**Keywords:** cultural evolution, cognitive anthropology, ethnomathematics, knot theory, cultural transmission, cognition

## Abstract

Few cultural practices beyond language are as widespread as string figure games. Their global distribution and potential to yield insights into cultural transmission and cognition have long been noted. Yet, it remains unknown how or when this behaviour originated and to what extent shared motifs are signals of repeated innovations or deep cultural transmission. Here, we combined a global cross-cultural inventory of string figures with a novel methodology based on knot theory, which enables the unequivocal numerical coding of string figures. We performed a computational analysis of a sample of 826 figures from 92 societies around the world. Across these societies, we found 83 recurring string figure designs, some of which are regionally restricted while others display a global distribution. The cognitively opaque nature of string figure designs and their clear geographic distribution reveal processes of cultural transmission, innovation, and convergent evolution. Most strikingly, the global distribution of some figures raises the possibility of shared ancient origins.

## 1. Introduction

**Figure 1.**
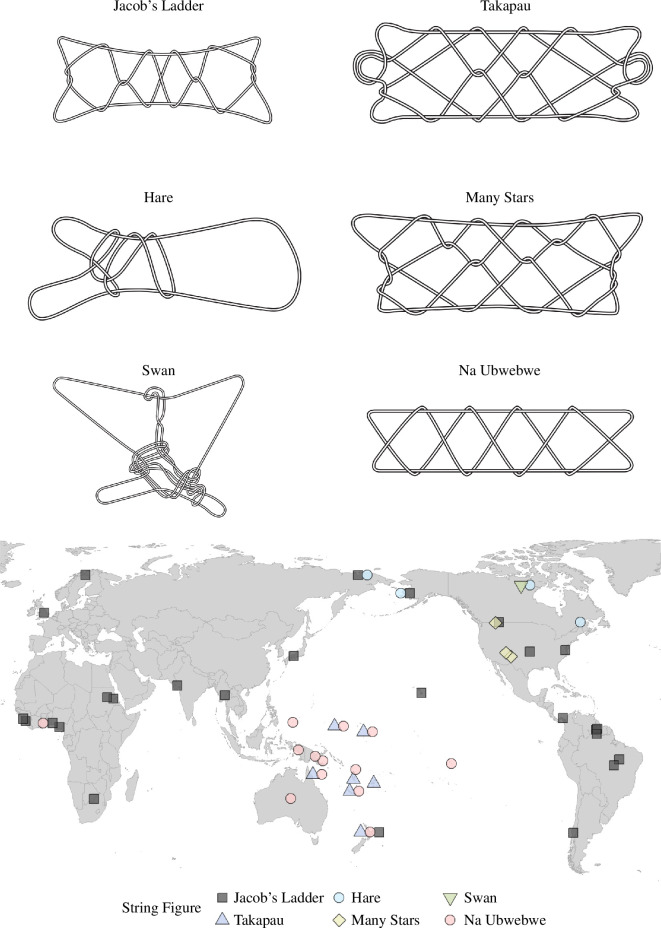
Six string figures with varying structural features and geographical distributions. *Jacob’s ladder*: one of the most widespread string figures (electronic supplementary material, 3). *Takapau*: a symmetrical string figure widely distributed in Oceania (electronic supplementary material, 3). *Hare*: an animated string figure common across the Arctic, representing a hare or caribou. The figure can be set to move as if it runs from right to left; it disappears when it reaches the left end. *Many stars*: a figure exclusive to western North America. *Swan*: a complex asymmetrical animal-form string figure documented among the Copper Inuit. Exemplary of Arctic string figure traditions, the *Swan* is associated with a story: the swan resolves into a loop when the maker releases it, representing a swan leaving its lake behind. *Na Ubwebwe*: a simple four-diamond motif, and perhaps the most widespread string figure structure in Oceania. Although it is made with various methods (electronic supplementary material, 3), its structure is practically exclusive to the Oceanic region. A map illustrating the geographical distribution of each of the six string figures in our dataset.

String figures are a practice found across cultures worldwide, involving the manipulation of a loop of string typically 1–2 m long. String figures involve transient designs made with hands and, less frequently, the mouth, wrists, and feet. They are among the most common forms of play and entertainment across cultural traditions worldwide. Beyond play, string figures hold significant importance in storytelling and origin stories [1–3], religious practices [1], divination [4,5], and even competitive mind sports [6,7]. Often, string figure designs are codified and named, and the act of making figures is integrated into the cultural transmission of traditional knowledge (figure 1). Notably, this is true of the practice at a global scale. The design of string figures is a cognitively demanding, procedurally opaque activity, and makes use of the ancient and evolutionarily important technology of cordage [8]. Few forms of material culture are shared this widely, making string figures a prime candidate to elucidate patterns and processes of cultural evolution, transmission, innovation, and deep ancestry.

Due to the impermanent and perishable nature of string figures, early evidence of their existence is scarce. An ancient Greek text refers to a string figure used two millennia ago [9], while the earliest direct evidence dates only to the seventeenth century [10,11]. In the ethnographic record, string figures are known by many names, from *ayatori* in Japan, *cat’s cradle* in the Anglophone world, *whai* among the Māori, to *ajarautit* among the Copper Inuit. Recognizing their analytical potential, scholars interested in human behaviour and culture have long studied string figures [12]. During the late nineteenth and early twentieth centuries, a veritable boom of string figure ethnography occurred when Western ethnographers, already familiar with the *cat’s cradle* game (first dated to 1768 [13]) from their childhoods [14], were intrigued to notice this tradition shared among the distant cultures that they studied. String figures proved a convenient way of establishing rapport in remote places [14,15]. Reminiscing on his travels in the Malay Archipelago between 1854 and 1862, Alfred Russel Wallace [16] wrote of one such unexpectedly familiar encounter:

One wet day, in a Dyak house, when a number of boys and young men were about me, I thought to amuse them with something new, and showed them how to make ‘cat’s cradle’ with a piece of string. Greatly to my surprise, they knew all about it, and more than I did; for, after I and Charles [Allen] had gone through all the changes we could make, one of the boys took it off my hand, and made several new figures which quite puzzled me. They then showed me a number of other tricks with pieces of string, which seemed a favourite amusement with them.

The discovery of similar string games, and the strikingly lookalike patterns found among them, inspired attempts to uncover their potential shared ancestry. Early anthropologists interested in string figures include luminaries such as Franz Boas [17], Alfred C. Haddon [14], Kathleen Haddon Risbeth [18], Knud Rasmussen [19], Julia Averkieva [20], Diamond Jenness [21], and Willowdean C. Handy [22], among many others [6,23,24]. The topic also attracted the attention of biologists [25,26], who sought to record string figures to test evolutionary hypotheses. Since cultures across the world were observed making similar or even identical figures, these were marshalled as evidence for common origins [27]. By this reasoning, string figures were used to infer cultural contact and as a means of marking potential migration trajectories.

During the peak of string figure interest, the topic garnered widespread scientific attention. Caroline Furness Jayne’s *String figures and how to make them* from 1906 included the analysis of dozens of string figures, accompanied by nearly a thousand illustrations [28]. Kathleen Haddon’s *Cat’s cradle in many lands* published in 1911 provoked favourable reviews not only in many established anthropological journals but also in *The Lancet* and *Nature*. Next to professional ethnologists, a number of practical string figure experts, such as Honor C. Maude [7,29] and the many citizen scientists of the International String Figure Association (ISFA), have contributed to a substantial corpus that documents diverse string figure traditions around the world [30]. In parallel with these documentation efforts, numerous ways of formally representing string figures have been developed. These range from Rivers & Haddon’s [14] pioneering efforts at writing a universal language for string figure construction to subsequent analyses of string figure structures and methods [31–34], some including mathematical notation [35,36].

The making of string figures has been linked to the development of mathematical thinking [32,37–40]. In the context of niche construction theory, it is now widely understood that toys that mimic tools play a critical role in the ontogenetic development of human cognition [41]. This has likely been the case for most, if not all, of human history. The oldest archaeological evidence for string is coeval with the emergence of behavioural modernity in *Homo sapiens* in Africa. Given the evident ubiquity and hence likely antiquity of string figure making, this behaviour may—as a ‘tool of the mind’ [42,43]—be implicated in the development of foundational human mathematical and spatial reasoning. As we argue below, this may have had downstream effects on the evolution of other cognitively demanding string technologies such as ropemaking, knotting, netting, and weaving.

As critiques of speculative cultural diffusionism were rising in the mid-1900s [44], researchers [45] became sceptical of using string figures as reliable proxies for cultural transmission and contact. Consequently, academic interest in string figures waned, partly due to a lack of methodological tools to analyse the accumulated data. None of the formal string figure notations offered so far has provided an unequivocal way to transcribe string figures in a manner amenable to global-scale computational comparative analysis. This gap is accompanied by a general lack of tools to formally study string technologies, which has hindered the ability to study their origins, spread, transmission processes, and cultural evolutionary significance.

To bridge this gap, we introduce a global, cross-cultural dataset along with a novel methodology based on Gauss code, adapted from knot theory. Our methods provide a way to transcribe string figures for computational comparative analysis. This opens new avenues for hypothesis testing in cultural evolution, such as studying mechanisms of cultural transmission, innovation, and deep ancestry. The introduced Gauss code methods are generalizable to other string-based technologies such as knots, braids, ropes, netting, basketry, and weaves, offering new analytical tools for studying these oft-neglected forms of material culture [46].

Following extensive manual transcription of string figures into Gauss code, we present the first global cross-cultural analysis of this ubiquitous phenomenon. We provide fresh insights into a persistent question: have similar string figures evolved by descent with modification from a common ancestor, or have they emerged independently as a result of convergent cultural evolution? Our analysis reveals patterns indicative of innovation and guided variation [47] within certain regions, while the global distribution of certain string figures strongly suggests a temporally deep shared ancestry reaching back, we suggest, into the Pleistocene.

## 2. Results

**Figure 2.**
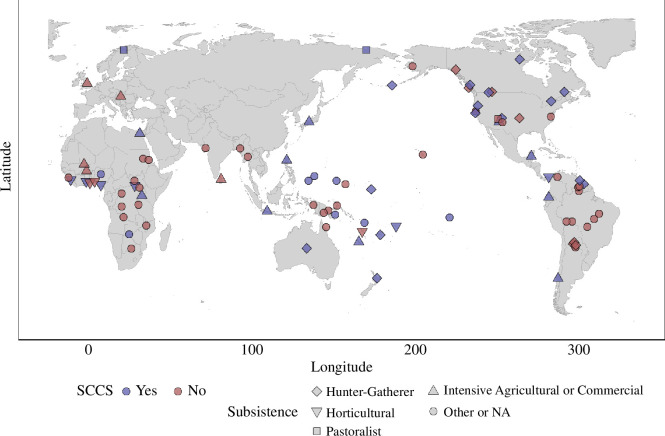
Cultures represented in our dataset. Coordinates are based on data from glottolog [48]. Cultures included in the standard cross-cultural sample (SCCS) are coloured blue, and other cultures are coloured red. The shapes of the pins vary by traditional subsistence strategy, illustrating how societies with variable economies have made string figures.

**Figure 3.**
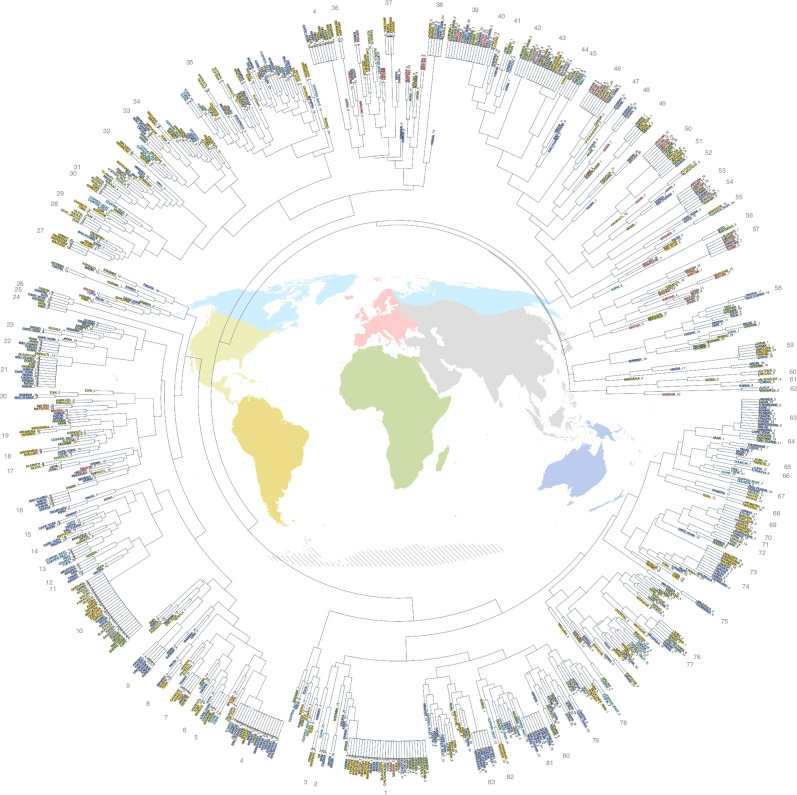
Dendrogram of the global string figure sample. Figure 3 is also available as a high-definition text-searchable PDF in the electronic supplementary material (figure S1) [49]. Individual string figures appear at the tips of the tree and are coloured by the geographical region (refer to the world map in the centre for colours). Higher order clusters group increasingly diverse figure designs. Clusters at the outermost edge of the tree contain identical or near-identical string figures. Clusters described in electronic supplementary material, table 1, are numbered and connected with a crossbar at the tip (the cluster numbers refer to the respective section in electronic supplementary material, table 1). Likewise, clusters close to each other (under the same branch) contain structurally similar string figures.

Applying knot theory (Gauss code) and string matching to the structural analysis of string figures, we found 83 classes of recurring string figure designs in a global sample of 826 string figures from 92 societies (figure 2; electronic supplementary material, table 1). The Gauss coding approach allows us to systematically quantify a string figure’s topological features (e.g. crossing order, overpasses and underpasses). Gauss coding of string figures has previously been proposed by Probert [35,36], although it was not used for comparative string figure analysis. We describe our specific approach to Gauss notation, which accounts for all possible ways of annotating a string figure with the Gauss code, in §4.2 and electronic supplementary material, 1, 2. This methodology allows comparative analyses and string matching. Using cluster analysis, we group identical or near-identical string figures together, with structurally similar string figures appearing in adjacent clusters (figure 3). Eighty-three designs appear in more than one cultural group and comprise a total of 380 individual string figures. Thus, 46% of the string figures in our sample have at least one identical pair; 188 or approximately 26% have at least five pairs; and 136 or approximately 16% have at least ten pairs. Electronic supplementary material, table 1, documents these in detail. In total, we identify 2035 pairs of structurally near-identical string figures with a cosine distance [50] of <0.1.

One major finding of this analysis is that people across highly culturally diverse, globally distributed societies practising a variety of different socio-economic strategies (figure 2) have made extremely similar string figures. While many scholars have observed this intuitively, this fact has hitherto not been convincingly demonstrated with a large-scale global dataset. Notably, *Jacob’s ladder-*class string figures are found on all continents, appearing in 26 cultural groups (cluster 1 in figure 3; see also figure 1). Other globally distributed string figures are the *Sun*/*Brokhos*-class string figures (cluster 4; see details in electronic supplementary material, 3 and table S1) and the *Two-diamond Jacob’s ladder* class figures (cluster 10). Electronic supplementary material, table 1 lists additional cases.

In contrast, some recurring string figure designs are regionally exclusive. The *Takapau*-class string figures (cluster 81) as well as the *Na Ubwebwe*-class string figures (cluster 63) appear in our dataset exclusively in Oceania (see also electronic supplementary material, 3, for an analysis of their construction methods). Similarly, the *Seagull* and *Hare* classes of asymmetrical string figures (clusters 78 and 13) are exclusive to the Arctic (figure 1). Most of the regionally specific clusters (total *n* = 49) are found in the Americas (*n* = 23) and Oceania (*n* = 18). Importantly, these are regions with relatively discrete ancient human dispersal histories. Electronic supplementary material, table 1, provides a detailed breakdown of regionally exclusive string figures.

**Figure 4.**
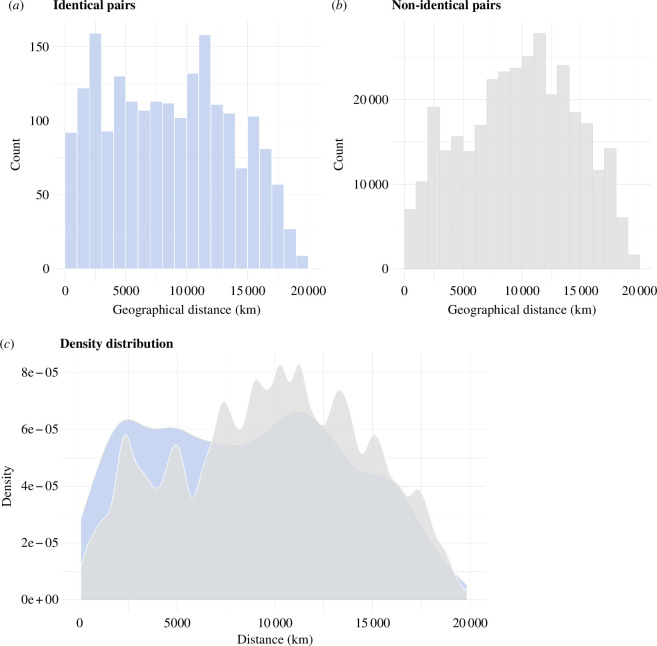
Analysis of the geographical distribution of structurally identical (*a*) and non-identical (*b*) string figure pairs. Histogram (*a*) (blue) depicts the geographical distribution of structurally identical string figures. Histogram (*b*) (grey) depicts the geographical distribution of non-identical (i.e. all other) pairs of string figures. Plot (*c*) overlays the smoothed density distributions of histograms (*a,b*) allowing the comparison of the geographical distribution of identical and non-identical string figures. Within-culture comparisons are excluded from this figure.

An analysis contrasting the geographical distribution of structurally near-identical (cosine distance of <0.1) and non-identical (i.e. all other) pairs of string figures shows that structurally identical string figures are more likely than non-identical ones to be found in geographically proximate social groups (i.e. groups with likely cultural contact; figure 4). However, some identical string figure designs can be found in very distant regions, e.g. the *Jacob’s ladder* (figure 1). We interpret this combination of regionally specific and widespread figures to indicate variable transmission histories. The discrete occurrence of particular and often complex designs only within specific regions suggests local innovation or invention and subsequent transmission between neighbouring and interacting groups. Conversely, the widely dispersed occurrence of some string figure designs reflects a deep ancestry not only of the practice in general but of particular designs (see §3 for elaboration).

String figure designs show their greatest similarity between adjacent and closely related groups, supporting the notion that proximity (the cultural contact it enables) is an important structuring element in cultural transmission. Similarly, the overall diversity of string figure designs attests to local experimentation and creativity, often with reference to local ecologies and environments. Our analysis also reveals a suite of globally distributed string figure designs that appear on every continent and that cut across ecological and socio-economic differences. The complex production processes and structures inherent in many of these widely dispersed figures suggest the potential for descent from a common ancestral template. In linking string figure designs to cultural transmission, we interpret this ubiquity as a signal of deep ancestry.

## 3. Discussion

### 3.1. Transmission or convergence?

The literature on the cultural transmission of string figures has ranged from claims of diffusionism—proposing that string figures share a common origin—to arguments that they evolved independently through cultural convergence. Using a large and unequivocally transcribed corpus of string figures, our analysis provides a data-driven perspective that finds noteworthy evidence for the high-fidelity cultural transmission of string figures, and of string figures that are shared globally.

Highly similar string figures are often shared by neighbouring groups (figure 4). Many string figure designs are geographically exclusive and only found within one cultural region. Using our clustering method, these regionally exclusive string figures can be confidently identified within the global dataset. Our data include two interesting groups of geographically restricted string figure structures. The first are the complex string figures that are unlikely to have been independently invented on multiple occasions. Although cross-culturally recurring string figures tend to be of the simpler kind, noteworthy exceptions exist (electronic supplementary material, table 1; figure S10). For instance, the relatively complex *Takapau* class (cluster 81) is exclusive to Oceania but widespread within that region (figure 1), suggesting common origins (see electronic supplementary material, 3, for further analysis of *Takapau*). Similarly, the *Many stars* and *Netted shield* classes (clusters 31 and 28) are shared exclusively by North American groups (figure 1). These cases illustrate how complex string figure patterns can be transmitted, even with high fidelity, between neighbouring societies and that geographic proximity and/or high levels of mobility play key roles in transmission (see also Paterson [23] on the transmission of complex Arctic string figures, and Ross & Atkinson [51] for similar features in folktales).

More difficult to explain but of considerable interest are those simple string figures that are, despite their uncomplicated structure, geographically exclusive. If string figures evolved convergently, one might expect simple string figures to be made the world over, but this does not seem to be the case. For example, the *Na Ubwebwe* class of string figures (cluster 63) is structurally simpler than the globally widespread *Jacob’s ladder*, although they are somewhat similar in overall appearance, both representing four diamonds in a row. Unlike *Jacob’s ladder*, *Na Ubwebwe* is virtually exclusive to Oceania. *Na Ubwebwe* design involves less common, wrist- or mouth-based opening moves, and a finish with the *Caroline Extension*, a trait seemingly exclusive to Oceania and especially common in Austronesian cultures (see electronic supplementary material, 4). We hypothesize that string figures like *Na Ubwebwe* and *Takapau*, which also appear in local origin stories [1], have deep Austronesian shared ancestry, with the constituent design features (including the *Caroline Extension*) having evolved around the time of early human dispersals in the region (see electronic supplementary material, 3). A similar case is the *Hare* class (cluster 13), exclusive to the circum-Arctic domain. The Arctic, too, is characterized by repeated prehistoric dispersals that have carried many shared cultural and genetic traits across the region [52–54]. String figures may have been among them. Cases such as these illustrate how string figure structures can travel unchanged across time measured in, at the least, centuries, and space measured in thousands of kilometres (figure 1).

Box 1. **Continuities and discontinuities in string figure transmission.**

Variations in the outcome (finished structure) and process (action sequence) of string figure designs can be used to address processes of cultural descent with modification [26]. The structures of *Takapau* and *Na Ubwebwe*, for instance, are exclusive to Oceania. Yet the methods for making them vary considerably. Our analysis (see electronic supplementary material, 3) suggests that *Takapau* is made with four different openings depending on where it is found, and *Na Ubwebwe* is constructed using six distinct methods. Despite these differences, it is evident that they are only found within societies with well-attested historical ties. In some instances, the figures even share the same names across vast distances, likely representing cultural transmission combined with local intentional modifications or minor changes in technique brought about as a result of copying errors [47]. The cultural transmission of string figure ‘recipes’ can be accompanied by changes in technique, leading to either innovation of new production techniques or modification of existing ones, even when the final product is preserved accurately.

For example, an individual might learn a new string figure and remember its overall finished appearance but not the precise method of its manufacture. A skilled string figure maker could experiment and alter their method for reproducing that figure, perhaps using a more elegant technique, or utilizing the move-sets more prevalent in their own cultural repertoire [12]. If this new technique is successfully passed along and becomes part of a particular group’s repertoire, it can be classified as innovation. Slight mistakes may also be introduced during transmission. For example, Fijians (BAU_FIJIANS_11) and the Indigenous Australian peoples at Cape York (CAPE_YORK_16) are documented making an almost *Na Ubwebwe*-class string figure, with a very slight structural variation. This variation sets their version apart from their Pacific neighbours (note their subtle distance from cluster 63, figure 3). Another case can be found in the various animal figures found in the Arctic (e.g. in figure 3, the pair CHUKCHI_9 *Wild Reindeer* and COPPER_INUIT_7 *Porcupine*) that are nearly identical (and regionally exclusive) but have some minor structural differences. These examples demonstrate that string figure designs—like many forms of traditional material culture [52,55,56]—are not always transmitted with maximum fidelity, causing them to evolve over time.

Transmission chains may also break. String figures, like other forms of human knowledge, may not always be transmitted accurately due to ‘noisy’ transmission channels, caused by limits in working memory or other social and environmental factors that constrain our cognitive faculties. Like similarly complex forms of material culture [57], string figures are cognitively demanding, and the precise details of their production are easily forgotten. Our dataset contains various possible cases of broken transmission chains: some moderately complex Melanesian string figures in our dataset (WAMPAR_10; TOLAI_7) are labelled with reference to the Sun and/or the Moon. These figures have a circular shape in the middle and a lozenge within. Eastwards and southwards in Melanesia, Micronesia and even coastal Australia, we find string figures with a simpler structure but similar overall appearance (of the common *Sun/Brokhos* class; see electronic supplementary material, 3 and table S1), also with names referring to the Sun and/or Moon (TIKOPIA_1, KIRIBATI_16, MALEKULA_13, CAPE_YORK_19). This same *Sun/Brokhos* class of figures is known also in remote Polynesia, but no longer by the same name. Matching these observations with known patterns of Austronesian migration [58], it is possible that this represents a broken transmission chain, where first structural details of string figures are lost, but labels remain, or vice versa.

Another potential case of broken transmission chains is found in the *Caterpillar* class (cluster 16). This figure is found throughout Oceania—labelled with very similar names, often referring to caterpillars or centipedes—yet the Tikopians make a figure representing a caterpillar (TIKOPIA_25) that while of superficially similar appearance, is based on an entirely different structure. Consequently, the Tikopian caterpillar does not locate near the *Caterpillar* cluster (cluster 16). This figure represents a case of motif emulation where the final appearance but not the underlying techniques have been transferred [32].

### 3.2. The case of *Jacob’s ladder*

Against the regional distribution of some string figures, the cross-cultural distribution of the *Jacob’s ladder* design stands out as a striking feature that demands closer inspection (figure 1). In addition to its global distribution, three further reasons indicate the shared and hence possible ancient origin of *Jacob’s ladder* designs.

First, despite its commonality, *Jacob’s ladder* is not an especially simple string figure. A useful outcome of representing string figures with numerical strings (Gauss code) is that they can then be measured in terms of complexity, accounting for how simple (number of crossings) and symmetrical or repetitive (algorithmically compressible) the design appears to be. Compared to a simple string figure, describing the structure of a complex figure will require more information. Our complexity analysis (see electronic supplementary material, 2 and table S1) assigns *Jacob’s ladder* class figures a structural complexity metric of approximately 3.8, which is well above our sample’s median score of 2.11. Regarding construction methods (electronic supplementary material, 3), *Jacob’s ladder* is finished with an unusual extension move that is not common among other string figures—a move that novices often find unintuitive and difficult to execute [31].

Second, in contrast to other widespread string figures such as the *Sun/Brokhos* class, the methods of making *Jacob’s ladder* are notably congruent globally. Our analysis of *Jacob’s ladder* class figures demonstrates that, remarkably, most *Jacob’s ladders* are made with precisely the same method (electronic supplementary material, 3). In fact, we identify only three distinct methods of making a true *Jacob’s ladder* in our global sample. Of these three, two are similar enough to be considered identical, and the third differs only in its opening. In contrast, the globally distributed simpler *Sun/Brokhos* is made with 13 distinct methods, most of which are markedly different.

The most common method to make *Jacob’s ladder* uses a common opening move for string figures called *Opening A* (see online resources [59] for descriptions of common moves). This is of particular interest here since *Opening A* is ubiquitous worldwide (see electronic supplementary material, 4) and is verifiably at least two millennia old. The first century CE [9] Greek physician Heraklas provides instructions beginning with *Opening A* for the construction of a medical sling, identical in structure to the *Sun/Brokhos*-class string figure (cluster 4) [60].

The subtle details of *Opening A* warrant closer inspection. *Opening A* is most often executed right hand first—indeed, not one of the *Jacob’s ladders* in our sample is created left hand first. Yet, there is no topological rationale for preferring the right hand, since a left-hand-first procedure (called *Opening B*) would follow the exact same protocol and result in a nearly identical structure. Innate human handedness bias may have some impact here, but note that with *Opening A*, the same move is repeated with both the right and left hands. Consequently, a right-hand-first *Opening A* is no easier for a right-handed person than a left-hand-first *Opening B*. Furthermore, *Opening A* is most commonly conducted with the index finger. Of all *Jacob’s ladders* in our dataset, only one (JAPAN_17) is made with the middle finger (*Opening Am*). Arguably, there is no practical reason to favour the index over the middle finger.

We suggest that if these designs were independently innovated, they should display considerably more diversity in production sequence. As such, the congruence of the methods of making *Jacob’s ladder* may be indicative of descent, occasionally with modification, from a common ancestor. Further experimental work could be designed to study how readily naive individuals innovate the *Jacob’s ladder* design or related moves.

Finally, the true *Jacob’s ladder* design is unlikely to be invented by chance, especially given the relative lack of lookalike figures. There exist many potential string figure structures similar to a *Jacob’s ladder*—let us label them *pseudo-Jacob’s ladders*. For instance, ZANDE_5 and KANAK_18, which both cluster near *Jacob’s ladder* (cluster 1) in figure 3, can be considered *pseudo-Jacob’s ladders*. They are near perfect lookalikes and would produce an identical shadow. Yet none of these *pseudo-Jacob’s ladders* display a wide geographic occurrence. The potential design space of *pseudo-Jacob’s ladders* is vast, but that space is very sparsely populated in our global sample. This speaks against true *Jacob’s ladders* occurring purely by chance in so many different places or having evolved through independent invention (see electronic supplementary material, 3). Rather, the true *Jacob’s ladder* figures are likely the result of social learning and cultural selection, which maintained this exact string figure form across generations [61].

We, therefore, contend that the ubiquity, complexity, and congruence of *Jacob’s ladder* across cultures combined indicate a shared and likely very old ancestry. We hypothesize that *Jacob’s ladder*, whose construction involves globally occurring moves such as *Opening A* and *Navajoing* (electronic supplementary material, 4), may be an ancestral state from which various other string figures have evolved. An objection might be that *Jacob’s ladder*, despite its commonality, is not present in many societies; however, we would caution that documentation of string figures across societies is highly incomplete (see §4.1) and we cannot expect to have a complete corpus from any society. Another option is that a small early corpus of string figures, such as *Jacob’s ladder*, the *Two-diamond Jacob’s ladder*, and the *Sun/Brokhos*, acted as ancestral states. The persistent association of string figures with storytelling supports the probability of their deep history, given the prevalence of string figure use in the oral traditions and origin stories of diverse societies with proven long oral histories [62,63] (electronic supplementary material, 4).

### 3.3. String figures in the history of string

The deep history of string figures remains poorly known. Besides the singular first-century Greek reference, the earliest direct evidence of string figures comes from seventeenth-century Japan, from literary sources [10] and a haiku [11]. String figures are, by definition, impermanent objects, and string—being made of natural fibres—is poorly preserved in the archaeological record. String is, however, omnipresent in human cultures. The earliest indirect evidence of string dates to 200−120 ka BP, with perforated objects such as ostrich eggshell beads intended for suspension [64,65]. Importantly, bead suspension implies the existence of a tied loop of string or string-like material (e.g. sinew), the only requisite material technology for a string figure. The oldest direct evidence of string is represented by a preserved cord made by Neanderthals dating to approximately 41−52 ka BP [66]. Coincident with the African Middle Stone Age, cordage was likely ubiquitous and used in the manufacture of a wide array of weapons, instruments, and facilities [67]. Since that time at least, the string has been essential to human ways of life. It provides material for garments, dwellings, nets, bowstrings, ropes, and many other technologies. String-making and use were time-consuming activities in preindustrial societies. Weaving even a quotidian item such as a basket could easily employ a single person for weeks [68]. The deep past of humankind is thoroughly intertwined with cordage, and the diverse materials that they can be used to produce are a suite of integral human technologies with diverse functions, some of which may be associated with string figures.

One such function is knotting [46]. Some string figures, such as the already discussed *Sun/Brokhos* class, have knot-like properties: they transform one open loop of string into four loops when tightened, forming a knot in the middle. This property is also present in many other common figures. It is plausible that figures with such properties may have been useful as quotidian knots and further work should seek to uncover the precise relationship between knots and string figures [46]. Notably, Polynesian gourd container nets also bear considerable resemblance to string figures, although unlike string figures they form a non-trivial mathematical knot and do not resolve into a loop [69–71].

In line with such functional considerations, it has also been suggested that string figures may derive from fishing technologies such as seine nets [72]. Interestingly, both fish and nets are common themes among the string figures in our dataset, with the fish-related concepts appearing in the name of 51 string figures. Since the fishing practices of all 186 standard cross-cultural sample (SCCS) cultures have been documented [73], we can compare the 42 SCCS cultures in our dataset with the remaining 144 SCCS cultures in terms of fishing subsistence. Using a fishing scale [73] from 0 to 10, the cultures in our sample have a mean score of 2.36 (s.d. = 1.94), while the other 144 cultures have a mean score of 1.44 (s.d. = 1.60). Although this comparison does not imply a causal link between fishing and the presence of string figures, it is noteworthy that the cultures documented making string figures tend to have a higher involvement in fishing compared to others. From ethnographic reviews of play, it is evident that play often involves mock versions of real tools, since toys often serve as learning aids, helping youngsters to learn vital technologies [74,75]. In this respect, string figures may be considered qualifier versions of everyday objects like seine nets. That said, string figures and nets [46] are ultimately made with very different methods, and the main benefit of string figure making for practices such as netting would be restricted to developing general skills like manual dexterity and spatial awareness.

String figure making may also have developed in the context of crafting technologies such as weaving, knitting or spinning [72]. In the Japanese *ayatori* tradition, many concepts related to string figures—including the word *ayatori* itself—derive from weaving or knitting [76]. String figures in Japan were traditionally made during the colder seasons, by children when parents were knitting [76], and in many other cultures, too, string figure making was a seasonal activity. This could be related to weaving, knitting or spinning often being indoor activities conducted predominantly during cold rainy seasons (electronic supplementary material, 4). Parents may have handed loops of string to children with the intention to not only preoccupy them but also to develop their manual dexterity or spatial cognition. This would eventually be useful for string manipulation. That said, our data overwhelmingly suggest that string figures are not exclusively child’s play (electronic supplementary material, 4).

However, we contend that string figures are not necessarily derivative of other string technologies, and we should consider seriously the alternative that the opposite is true. For one, weaving, basketry and net-making are all technologies requiring a great deal more equipment and much longer processing chains compared to the making of string figures (which only require a loop of string or sinew and the makers’ own appendages). Accordingly, the making of string figures may be one of, if not the, earliest forms of string manipulation. A simple and perhaps multi-purpose loop of string is easy to invent and portable, as is illustrated by the many cultural groups who wear loops of string as decorative garments such as bracelets or belts, or for any number of utilitarian purposes (electronic supplementary material, 4). Such portability was a vital characteristic for any early technology among the mobile hunting and foraging societies of the deep past. Thus string figures would have been accessible forms of entertainment (e.g. as visual aids for storytelling) even before the advent of container technologies [77].

### 3.4. String figures in human cognitive evolution

The ubiquity of string figure making across diverse cultures prompts exploration into its underlying causes. One straightforward explanation is the combination of two factors: the fundamental role of play in human development [41,78] and the sheer availability of string in material traditions. Play is a universal characteristic of human culture, serving important developmental functions, and expanding our creativity in material engagement [79]. Games and pastimes like string figures allow individuals to freely practice manual dexterity and cognitive skills, which, along with the cultural transmission of these skills, have clear evolutionary advantages. A simple loop of string is cheap and readily available in practically all human societies, making it an ideal tool for play across all ages. This accessibility, coupled with the importance of play, may be sufficient to account for the widespread popularity of string figure games.

Alternatively, especially the commonality of specific string figure designs could have resulted from so-called cognitive ‘attractors’ [80]. In this view, certain features of both string loops and human bodies or minds create affordances that explain the recurrence of specific cultural patterns [81]. Our shared bodily, cognitive, and perceptual capabilities have resulted in the independent cultural evolution of similar units of measuring [82] and counting [83], similar numerical systems [81], as well as stellar constellation categories [84]. Arguably, there may simply be something aesthetically pleasing about net-like imagery that resonates with our visual system [85]. Hence, there could be a cognitive predisposition in humans to invent or readily adopt (especially net-like) string figures. Human anatomy (our ten fingers) constrains string figure making, although the mouth and feet do offer ways of extending the total design space. String figure topology also dictates that some figures emerge more readily than others [72]. That said, the total design space of all humanly possible string figures is likely far greater than that which the known string figure corpus populates.

**Figure 5.**
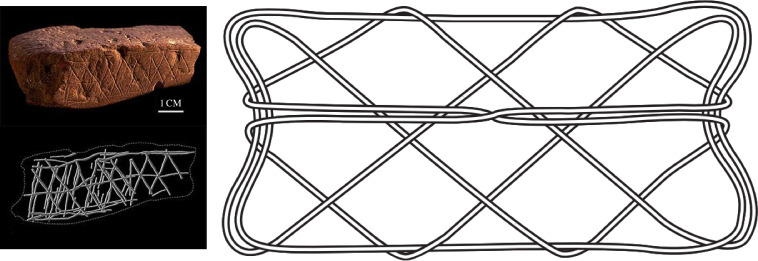
Net-like diamond patterns are common themes in prehistoric art. Some net-like patterns resemble common string figure structures. Although this does not indicate that these prehistoric patterns represent string figures, they do demonstrate the ancient human visual appeal for such motifs. Left: Ochre stone art found at the Blombos Cave site, South Africa (*ca* 70 000 BP) (Wikimedia Commons, by Chris S. Henshilwood, CC-BY-SA−4.0). Right: A string figure of the Polynesian *Turtle shell* class.

While fragmentary, the archaeological record may offer hints regarding the antiquity of string figures. Net-like representations reminiscent of string figure designs are common in the artistic record across time and space. For example, in Europe, depictions of net-like figures, as well as ropes and ladders, are common in prehistoric rock art [86], and net-like figures appear in rock carvings of the European Bronze Age [87]. Ladder-, fence-, and basket-like motifs are common in European Upper Palaeolithic cave art [88] and may even extend to a possible 64.8 ka BP Neanderthal ladder-motif at La Pasiega Cave in northern Spain [89]. String figure-like patterns are also documented in Bell Beaker pottery (fig. 4:2 in [90]) from approximately 4 ka BP. Most notably, the earliest known engraved art from Blombos Cave in South Africa made by early *Homo sapiens* at approximately 73 ka BP [91] depicts a pattern remarkably similar to common net-like diamond string figure designs in our dataset (figure 5; cf. also *Jacob’s ladder* and other motifs in figure 1). In line with other recent cross-cultural explorations of poorly preserved material culture—children’s toys [74], clothing [92], clubs, and throwing sticks [93]—it seems likely that the wide distribution of string figures, along with the primacy of string as a Palaeolithic technology [8,94], suggests that they may be deeply woven into the fabric of human evolution.

If string figures indeed have deep ancestry, they can be interpreted as an early form of mathematical thinking. Studies on ethnomathematics typically focus on numeration systems [81] and refer to the use of weights and accounting systems [95]—developments that occurred relatively late in human evolution. Recent work also points to possible Palaeolithic accounting technologies involving pebbles [96] and hand stencils [97]. However, mathematical thinking is not restricted to discrete mathematics or other fields with well-documented histories (e.g. geometry). Far less attention has been paid to early topological cognition.

Topology, a well-established branch of mathematics, studies properties preserved through continuous deformations like stretching, bending and knotting—the very fundamentals involved in creating string figures [32]. String figures engage with a variety of topological concepts: by manipulating string to form and resolve knots, practitioners effectively explore the mathematical state space associated with knot theory. This hands-on practice parallels topological proofs, which often rely on visual representations (e.g. Reidemeister’s theorem), making string figures practical demonstrations of topological relationships and transformations. If we acknowledge topology as a legitimate branch of mathematics—which we certainly should—then the practice of creating string figures is placed squarely within the realm of ethnomathematics, or more precisely, we suggest, ‘ethnotopology’. This recognizes string figures not merely as cultural artefacts but as tangible explorations of early mathematical ideas.

The creation of complex string figures demands various kinds of mathematical thinking, including algorithmic reasoning, spatial visualization, and counting. While simple string figures can be learned through imitation, complex figures often present unpredictable puzzles that require topology-savvy mental simulation and a deep understanding of string behaviour. This is especially true of their original invention. For example, the Inuit ‘whale and fox’ [28] figure involves merging and disentangling two distinct animal shapes into one string figure, demonstrating practical mastery of topological cognition. Many cultures have developed systematic algorithms for creating string figures. The Nauruan *Eongatubabo* [7] sequence—a complex ten-step sequence that can be used to generate a wide repertoire of figures—exemplifies explicit algorithmic thought. Notably, this ‘algorithm’ even has a local name, not an unusual feature among string figure traditions [22]. Constructing intricate figures also often involves counting and keeping track of loops and twists, engaging with discrete mathematics. This emerging ethnotopology, along with the manual dexterity it develops, may have catalysed the evolution of other essential string technologies such as knotting, netting, and weaving, which also follow distinctively algorithmic operation chains [68].

Material culture scaffolds have a critical impact on the emergence of mathematical cognition, and we here extend this argument to include the ethnotopology of string figures. Cross-culturally, humans have not settled at merely making simple string figures. The most complex string figures in our dataset have up to 89 crossings. They form elaborate topological and geometric forms, and some even have animated properties (see figure 1 caption). Commonly, even a complex figure may resolve into an open loop simply by pulling from two parts of the string. Around the world, diverse societies have created elaborate string figures whose design requires tremendous diligence, sustained attention, and, we argue, mathematical cognition.

We contend that string figures reflect a deep human cultural evolutionary history of material engagement, innovation, and creativity [98]. Regionally distinguishable traditions go hand in hand with a distinct repertoire of globally distributed, shared designs, such as *Jacob’s ladder*, whose origins may lie deep in the Pleistocene. The original engagement with strings at this time, and its subsequent global dispersal alongside *Homo sapiens* could have catalysed a specifically human mathematical reasoning. Even today, in some cultures, string figures are presented as puzzles to be elaborated upon [7]. Often, string figures play an active role in storytelling and hence in the maintenance of traditional knowledge (electronic supplementary material, 4). Occasionally, the making of string figures has even appeared as a form of showmanship, whereby skilled string figure making entails prestige or demarcates talented individuals from others, and where string figures take on a status as a kind of competitive mind game [6,7].

Cross-cultural analyses of human behaviour and technology are a powerful way of inferring their cultural evolutionary history [99–101]. Few such behaviours can be coded unequivocally, however, and many technologies are unevenly distributed globally. We have here presented a formalized corpus of string figures from societies around the world with varying socio-economic strategies, occupying very different habitats and possessing diverse cultural histories. Our novel approach to annotating these figures using Gauss code allows for the identification of design similarities and this in turn reveals that some designs occur only within certain cultural regions, while others are shared across many societies around the globe.

Future studies could combine linguistic, genetic, or other cultural data with information on string figure designs in, for instance, the Arctic or Oceania, to infer the co-evolutionary dynamics of genes, language, and material culture. We are currently developing an extension to the present method that will formalize the process of making a string figure, thus allowing the full and complementary computational analysis of string figure structures and construction methods [see also 31,34]. Cross-cultural experiments could further explore to what degree physical and cultural predispositions determine string figure production—for example, exploring how transmission biases [61] or transmission isolating mechanisms may affect the pattern of string figure distributions. Mathematical modelling could assist in better defining the total design space available. Such work is as timely as it is urgent. Many ethnographers already recognized a century ago [22,26] that traditions of string figure making across the globe are rapidly disappearing. Despite their global prevalence, string figure traditions are at acute risk of cultural extinction. In this context, the digital curation and analysis of string figure data as presented here serve to safeguard this remarkable shared human cultural heritage.

## 4. Material and methods

### 4.1. Data collection

The bulk of pertinent string figure literature has already been compiled into a regionally organized bibliography [30]. We use this bibliography in collating a globally and culturally distributed sample of string figures. Data collection was based on the following procedure:

1. We used resources such as the ISFA’s regional bibliography to find illustrations or photographs of string figures. These were then coded numerically using an application of Gauss code. Only string figures that are Gauss-codable were considered (see §4.2 and electronic supplementary material, 1 and 2, for details on our Gauss coding protocol).
2. For each cultural group, we collected a maximum of 25 string figures. This cap was introduced for practical reasons. Most cultures in our dataset have under 10 documented string figures (electronic supplementary material, figure S2). In contrast, a select few cultures have especially large string figure repertoires. To avoid skewing our dataset, and to limit the time-consuming work related to data collection (see §4.2), we stopped collecting string figures from one culture after we had successfully coded 25 string figures (starting from the beginning of each document). Cultures where we had to apply the limit of 25 figures included the Japanese, Ngombe, Copper Inuit (Inuinnait), Yup’ik, Kwakwa̱ka̱ʼwakw (Kwakiutl), Tikopians, Kanak, the Indigenous Australians from Cape York, Hawai'ians, Mapuche, Toba, Warao, and the British.
3. In principle, we only coded string figures whose making was recorded before 1990. We surmise that the nature of cultural transmission has been radically altered by the Internet. Therefore, assessing similarities of string figures recorded after the Internet age would bias some cross-cultural comparisons.
4. Some string figures appear in series or sequences, where one string figure is seamlessly transformed into another (e.g. the I-Kiribati Te Wau series in [1], or the British cat’s cradle series in [102]). In those cases, we coded all stable forms of string figures that were attributed with cultural meaning (i.e. figures within a sequence that are individually named or considered to represent something separate from the preceding or following figure).
5. In the regions of Oceania (Melanesia, Micronesia, Australia, and Polynesia), Europe, and the Arctic (from Chukotka to Greenland), string figures have been documented in such abundance that we had to impose limits on data collection. In these regions, we prioritized data collection to cultures in the SCCS (the SCCS is a cultural sample designed to include relatively independent cultures from each cultural region). For all other regions, we documented all string figures based on the above criteria.

The sample investigated in the present study consists of 826 string figures from 92 cultural groups (figure 2). In the future, we intend to expand the study to include the entire string figure corpus. We note that in the likely near future, such analyses can be greatly facilitated by computer vision and artificial intelligence. Other metadata collected include: the geographical coordinates of each culture obtained from glottolog [48], bibliographic metadata, outline of world cultures (OWC) culture codes, glottocodes (linguistic data), coverage dates, traditional subsistence types, as well as the names of the string figures themselves (in both the local language and translated into English where available).

It is unlikely that we have a complete documentation of any culture’s string figure repertoire due to two primary factors. First, by the time of early ethnographic documentation, string figure traditions were already in decline. This pattern of cultural erosion, often linked to colonial influences and the broader loss of oral traditions, is evident across many societies. For example, Fijian string figures were observed as being endangered in the early twentieth century [26] and similar themes were noted in Palau [103], Japan [76], and among the Māori [6] and Nuxalk [2]. By the time anthropologists documented these practices, many figures had already likely been lost.

Second, games and play, which include string figures, are generally under-documented in ethnographic records [78]. Despite generating much interest among early scientists, string figure traditions were typically collected from just a few informants and often as a secondary interest during fieldwork on other better-funded topics. Consequently, we cannot assume that these accounts represent the full corpus known to a society at a given time. Academics like Evans-Pritchard [104] and Mary and Louis Leakey [15] have noted that string figure traditions were sometimes completely overlooked by other ethnographers. Contributing to further data incompleteness, since the 1950s, academic research on string figures has declined, with most knowledge now preserved by citizen scientists (especially at ISFA) and traditional and indigenous practitioners. As a result, the prevalence of any specific string figures in the present dataset should be interpreted as a lower bound estimate.

With these caveats in mind, to assess the prevalence of string figures across the globe, we may use the SCCS, a sample of 186 relatively independent world cultures. Our dataset contains 42 societies from this sample, indicating that string figures are confirmed to have been made in at least 22.6% of the SCCS (and so, in over a fifth of world cultures). This is strictly a lower bound estimate, and the true prevalence is likely to have been more common. For example, there is a notable lack of documented string figures in regions like Central Asia and northern Africa, which may merely reflect the sparse ethnographic research conducted in these regions.

### 4.2. Methods

#### 4.2.1. Gauss coding

Each string figure in our sample is formally annotated using Gauss code, a method from knot theory used to concisely and systematically represent and analyse knots in mathematical terms [105]. Gauss coding involves assigning numbers to the crossings of a knot diagram (in this case, a representation of a string figure on a two-dimensional plane) and then describing the interlacing structure of the string by a sequence of these numbers. This encodes the string figure’s topological information.

In our Gauss coding scheme, each crossing in a string figure diagram is labelled with a positive integer, and whether the string passes over (+) or under (−) at a given crossing determines the sign of the integer in the sequence. Gauss code is constructed by following a defined path along the knot diagram and recording the labels of the encountered crossings. This sequence of numbers represents the string figure, allowing for comparisons, computations, and the study of various string figure properties. We describe our specific application of Gauss coding more precisely in electronic supplementary material, 1,2.

Probert [35,36] first applied Gauss coding to assess whether or not a string figure diagram would resolve into an open loop. Storer [31] also devised a method of string figure formalization (the ‘linear sequence’) that includes aspects similar to Gauss code. A universal property of string figures is that they are entangled *unknots* (or *trivial knots*) and as such resolve into a loop. That is, every string figure is made from a loop of string, and any manipulation done to create a string figure preserves the topological structure of the knot, so it must remain unknotted. This is a useful property for assessing the correctness of string figure Gauss code, since methods from knot theory may be used to test whether or not a given string figure diagram is truly unknotted. Such an application is available in Probert’s String Figure Analyser (hosted by ISFA), which we use to assess the validity of our Gauss codes [35,36].

Besides requiring considerable manual labour, standard Gauss coding has a few notable shortcomings for the analysis of string figures. First, although all string figures can theoretically be represented by a Gauss code, not all string figures are conveniently represented in this way. The highly three-dimensional string figures are especially problematic since their depiction in a two-dimensional diagram may result in ambiguities. This is also true of the most densely entangled string figures, whose structural properties (over- and underpasses) are difficult to decipher. See electronic supplementary material, 1, for a discussion of these limitations.

Further, defining only one Gauss code does not reliably allow the matching of two similarly structured string figures. This is because, when a string figure is assigned a single Gauss code, the basepoint (the point of the string where one starts labelling the Gauss code) and orientation (the direction of notation, clockwise or counterclockwise) of this code are arbitrary. This means that there are many ways to annotate one string figure with a Gauss code, and these Gauss codes can appear quite different from each other, all the while encoding the same information (electronic supplementary material, 2). The upshot is that a standard Gauss coding method would not suffice for a cross-cultural string figure comparison, since similarly structured string figures may be held or presented, and thus coded, in many ways.

To solve the problem of arbitrary basepoint and orientation, and to ensure that two identically structured string figure diagrams can be identified no matter how they are Gauss-coded, we developed an algorithm in the programming language R, *GaussCodeR*. When it is given one Gauss code as input, *GaussCodeR* generates all possible Gauss codes that the string figure diagram could be represented with. *GaussCodeR* is explained in natural language in electronic supplementary material, 2, allowing its reproduction in other programming languages.

Consequently, each string figure diagram in our dataset is represented numerically with an integer string that includes all possible Gauss codes it can be described with. This sequence of Gauss codes (the ‘Gauss code sequence’) is typically thousands of integers long. Each sequence models the topological structure, and importantly also the substructures of its associated string figure. This allows for the matching of two or more identical or highly similar string figures. The method also accounts for mirror images and perspective (i.e. whether the figure is represented from the viewer’s or the maker’s perspective). In effect, this integer string can be considered the ‘DNA sequence’ of a string figure: it encodes the string figure’s structure in a string of symbols, allowing its matching and comparison with other string figures. The Gauss code sequence of each string figure may also be given an information theoretical metric to quantify its structural complexity, using compression algorithms, as explained in electronic supplementary material, 2.

#### 4.2.2. String matching and clustering

Once each string figure is represented with a Gauss code sequence, they can be compared using string-matching algorithms. We use q-grams [50] for Gauss code sequencing. The q-gram method is especially appropriate here since it allows the identification of string figure substructures. This enables the identification of not only identical string figures but also string figures that share structural properties (over- or underpass sequences of length *q*). The q-gram is a technique used in computational science to represent textual data as fixed-length substrings of a given length, denoted by *q*. We use *q* = 3 to analyse string figures since we consider a three-crossing substructure to be the structure of minimal interest in a string figure (a *q* between 2 and 5 will bear similar results, with higher *q* values being more conservative). A 3 gram would break the Gauss code ‘1 −2 −3 4 5 −6 2 −1 −7 −5 −4 3 6 7’ into a set of all possible sequences of length 3, {‘1 −2 −3’, ‘−2 −3 4’, ‘−3 4 5’, ‘4 5 −6’, …}, thus recording the topological *substructures* of each string figure. Each string figure is considered to consist of various substructures: two identical string figures will have an identical set of substructures and two similar string figures will share many substructures.

Using the R package *stringdist* [50]*,* we create a q-gram profile for each string figure. Each substructure in the string figure’s Gauss code sequence is thus encoded in a structural profile that can be compared with other string figures. We then compare these q-gram profiles by taking their cosine distance (see *stringdist* [50] documentation for details). From this, we generate a cosine distance matrix where each string figure in our dataset is compared to the rest. This assigns pairs of string figures with a metric of structural (dis)similarity (cosine distance) that ranges from 0 (identical) to 1 (completely different). Thus, string figures that share many substructures will appear similar (a low cosine distance), and identical string figures (with all the same substructures) have a cosine distance of approximately 0.

Finally, using the cosine distance matrix we visualize the entire string figure dataset using a hierarchical clustering algorithm and produce a dendrogram. Working in R, we use the hclust() function under complete linkage clustering. The complete linkage method creates various small and discretized clusters, under the assumption that a single and real existing object represents each cluster [106]. This is apt for our purposes, since we may assume that a cluster is represented by a real, material, string figure, and we are interested in visualizing many discrete clusters of structurally variable string figures. For data visualization, we use *ggtree* [107] to generate a circular dendrogram. The dendrogram illustrates the sequence of cluster fusion and the distance at which fusions occur from each other.

Since we are interested not only in the similarities between string figures but also in their geographical distribution, we calculate the geographical distance of each string figure pair in our dataset (based on geographic coordinates). We calculate geographical distances using Haversine distance, creating a geographical distance matrix that is comparable to the cosine distance matrix. This allows the analysis of the geographical distribution of similarly structured string figures.

## Data Availability

All data and R code used for analysis are available at the OSF repository for this paper [108]. Supplementary material is available online [49].
